# Editorial: New immunotherapy strategies and related therapeutic targets for gastrointestinal malignancies

**DOI:** 10.3389/fimmu.2024.1389781

**Published:** 2024-03-08

**Authors:** Ganesan Ramamoorthi

**Affiliations:** Clinical Science & Immunology Program, H. Lee Moffitt Cancer Center, Tampa, FL, United States

**Keywords:** cancer immunotherapy, dendritic cells, immune checkpoint inhibitors, gastrointestinal malignancies, prognostic biomarkers

Malignancies in the gastrointestinal tract, including esophagus, stomach, colorectum, pancreas and liver, are among the most common cancer types and pose severe health challenges in patients. Current treatment options for gastrointestinal malignancies includes surgery, chemotherapy, radiotherapy, and molecular therapeutic targets. Although chemotherapy combined with molecular therapeutics are typically used for patients with advanced stages of gastrointestinal malignancies, only limited benefits have been observed. Cancer immunotherapy, including immune cell-based therapy, has been emerging as an effective therapeutic approach for treating various cancers including gastrointestinal malignancies. However, the potential targets for developing immunotherapy and prognostic biomarkers that predict treatment response have not been extensively investigated in gastrointestinal malignancies. This Research Topic comprises a series of original research articles and state-of the-art reviews that focused on identifying and summarizing the most promising targets for developing immunotherapies and identifying potential biomarkers that may predict the patients that are likely to respond to immunotherapeutic approaches in gastrointestinal malignancies.

The immunosuppressive tumor microenvironment (TME) can prevent response to conventional therapies and various immunotherapies and lower tumor-infiltrating lymphocytes (TIL) in cancer patients. Wegierek-Ciura et al. have demonstrated that sequential peritumoral delivery of tumor antigen targeting dendritic cells in addition to anti-IL10R antibody with the immunomodulatory methotrexate-hydroxyethyl starch (HES-MTX) nanoconjugate unlocked an effective anti-tumor immune response with inhibition of various immune suppressive cells including T regulatory cells and tumor associated macrophages in a MC38 murine colon carcinoma model. This combination treatment enhanced tumor infiltration of CD4, CD8 and NK cells into the tumors and induced tumor regression. With the help of machine learning and a multiple omics bioinformatics approach, Zhu et al. have attempted to provide a molecular characterization for colon cancer and identified three clinically relevant subtypes and risk-related genes that biologically contributed to tumor aggressiveness, recurrence and metastasis in colon cancer patients. The authors have also extended their findings and confirmed differential expression status of PPARGC1A and GARBD genes in colon cancer specimens of patients and suggested these genes as potential therapeutic targets. Adaptation of a watch and wait (WW) strategy can be recommended for rectal cancer patients after achieving clinical complete response (cCR) treated with neoadjuvant chemoradiotherapy. This WW strategy can provide survival benefits similar to patients who underwent surgery, but it would prevent surgical trauma and preserve organ function in these patients. A retrospective study from Yang et al. compared the survival outcomes of dMMR/MSI-H locally advanced rectal cancer patients treated with neoadjuvant immunotherapy (anti-PD1 antibodies) who underwent surgery with confirmed as a pathologic complete response (pCR) versus treated patients opted for a WW strategy after achieving a cCR or near-cCR. After a median follow-up for 25 months, patients from both arms were free from local recurrence and distant metastasis with improved survival, suggesting that these patients may benefit from the WW strategy and surgery related complication can be prevented. The findings of this study need to be further validated and confirmed in longer follow-studies and prospective trials.

The reduced or loss of expression of SLC4A4 has been shown to promote cancer cell proliferation and metastasis in renal cancer cell carcinoma. Rui et al. explored the role of SLC4A4 in colorectal cancer and found that SLC4A4 downregulation was positively correlated with microsatellite instability (MSI) and associated with poor prognosis in colorectal cancer. This study suggests that SLC4A4 may be a potential prognostic biomarker with MSI to guide treatment for colorectal cancer. When comparing colorectal patients with dMMR/MSI-H, patients with pMMR/MSS may not achieve satisfactory response to conventional therapies and immune checkpoint inhibitors.

It is well known that combination therapy approach can combat cancer more effectively, reduce side effects and resistance and improve treatment outcomes in patients. Chen et al. in a review, highlighted the molecular aspects and recent advancements of various immunotherapy approaches and their potential synergistic effects with chemotherapy, radiotherapy and preoperative strategies for colorectal cancer. The authors listed various clinical trials that investigated these combination therapy approaches and examined their efficacy and safety in colorectal cancer patients. Immune checkpoint signaling activation can prevent anti-tumor CD4 and CD8T cell mediated immune responses in cancers. Over expression of the immune checkpoint protein CD276 (B7-H3) in cancer cells and tumor vasculature has been associated with tumor aggressiveness in pancreatic, hepatic and gastric cancers. Lutz et al. has developed a novel IgG bispecific antibody CC-3 (B7-H3xCD3 specificity) and confirmed its specific binding to B7-H3 expressed on pancreatic, hepatic and gastric cancer cells. This CC-3 bispecific antibody was able to inhibit cancer cell proliferation and induced CD4 and CD8T cell activation, proliferation, functionality and expansion of memory phenotypes, highlighting the therapeutic applicability of a CC-3 bispecific antibody to be further evaluated in preclinical and clinical studies.

Hepatic arterial infusion chemotherapy (HAIC) has been shown to improve the prognosis of patients with advanced liver cancer which opens a new opportunity to explore the combination strategy of immunotherapy and targeted agents with HAIC. Zhang et al. reported that combination of immunotherapy and molecular targets with HAIC increased tumor regression and surgical conversion rates in primary unresectable liver cancer patients. Lu et al. showed that the combination of the naturally occurring bioactive compounds quercetin, kaempferol, licochalcone A, naringenin, and formaronetin treatment were able to alter various intracellular signaling proteins (PI3K-AKT, p53 and VEGF) in the TME and inhibit progression of colorectal cancer in a mouse model. This treatment also inhibited PD-1 expression on T cells and increased cytotoxic activity of T cells leading to enhanced anti-tumor response in a colorectal cancer mouse model. Wang et al. reported that downregulation of SLCO1B1 as a prognostic signature and predicated overall survival of patients with hepatocellular carcinoma (HCC). Notably, authors have observed that the over expression of SLCO1B1 was able to inhibit the proliferation and migratory and invasive potential of HCC cells.

Oxidative stress and its associated signaling pathways have been explored to contribute to progression of gastric cancer and metastasis. Various oxidative stress-specific therapeutic targets and agents may be a promising strategy in combination with immunotherapy to effectively modulate the anti-tumor immune response and suppress pro-tumorigenic signaling pathways in gastric cancer (Liu et al.). Zhang et al. suggest that the prognostic nutritional index may be used as a prognostic biomarker of treatment outcomes in patients treated with immune checkpoint inhibitors for gastrointestinal malignancies. Immune cell-based therapies directed to target highly immunogenic tumor antigens will be critical for triggering stronger anti-tumor immunity. Ai et al. comprehensively summarized various cancer/testis antigens and their expression status in digestive tract cancers and highlighted potential immune cell-based therapies, targeted antibodies and an oncolytic virus-based therapy approach that can be effectively utilized to harness the anti-tumor immune response. Cancer risk related gene signatures and biomarkers for early esophageal squamous cell cancer (ESCC) diagnosis was explored by Ren et al. and found elevated expression of F2RL2 and reduced expression of SLC4A9, EXPH5, and MAGEC3 in tumors. The findings of this study suggest that these gene signatures may predict response to immunotherapy in patients with ESCC.

In summary, this Research Topic addressed new strategies and tumor specific targets to develop an effective immunotherapy including targeted antibodies, dendritic cell and adaptive cell-based therapies to harness the anti-tumor immune response in gastrointestinal malignancies. I hope that this Research Topic will provide new concepts for readers to further explore the potential role of these biomarkers in predicting treatment outcomes and new immunotherapies to treat gastrointestinal malignancies.

## Author contributions

GR: Conceptualization, Data curation, Writing – original draft, Writing – review & editing.

